# Application of Machine Learning and Weighted Gene Co-expression Network Algorithm to Explore the Hub Genes in the Aging Brain

**DOI:** 10.3389/fnagi.2021.707165

**Published:** 2021-10-18

**Authors:** Keping Chai, Jiawei Liang, Xiaolin Zhang, Panlong Cao, Shufang Chen, Huaqian Gu, Weiping Ye, Rong Liu, Wenjun Hu, Caixia Peng, Gang Logan Liu, Daojiang Shen

**Affiliations:** ^1^Department of Pediatrics, Zhejiang Hospital, Hangzhou, China; ^2^College of Life Science and Technology, Huazhong University of Science and Technology, Wuhan, China; ^3^Key Laboratory of Ministry of Education for Neurological Disorders, Department of Pathophysiology, School of Basic Medicine, Tongji Medical College, Huazhong University of Science and Technology, Wuhan, China; ^4^Key Laboratory for Molecular Diagnosis of Hubei Province, Tongji Medical College, The Central Hospital of Wuhan, Huazhong University of Science and Technology, Wuhan, China; ^5^Central Laboratory, Tongji Medical College, The Central Hospital of Wuhan, Huazhong University of Science and Technology, Wuhan, China

**Keywords:** WGCNA (weighted gene co-expression network analyses), SOM (self-organization map), aging brain, random forest, machine learning

## Abstract

Aging is a major risk factor contributing to neurodegeneration and dementia. However, it remains unclarified how aging promotes these diseases. Here, we use machine learning and weighted gene co-expression network (WGCNA) to explore the relationship between aging and gene expression in the human frontal cortex and reveal potential biomarkers and therapeutic targets of neurodegeneration and dementia related to aging. The transcriptional profiling data of the human frontal cortex from individuals ranging from 26 to 106 years old was obtained from the GEO database in NCBI. Self-Organizing Feature Map (SOM) was conducted to find the clusters in which gene expressions downregulate with aging. For WGCNA analysis, first, co-expressed genes were clustered into different modules, and modules of interest were identified through calculating the correlation coefficient between the module and phenotypic trait (age). Next, the overlapping genes between differentially expressed genes (DEG, between young and aged group) and genes in the module of interest were discovered. Random Forest classifier was performed to obtain the most significant genes in the overlapping genes. The disclosed significant genes were further identified through network analysis. Through WGCNA analysis, the greenyellow module is found to be highly negatively correlated with age, and functions mainly in long-term potentiation and calcium signaling pathways. Through step-by-step filtering of the module genes by overlapping with downregulated DEGs in aged group and Random Forest classifier analysis, we found that *MAPT*, *KLHDC3, RAP2A*, *RAP2B*, *ELAVL2*, and *SYN1* were co-expressed and highly correlated with aging.

## Introduction

The brain is highly sensitive to aging and lots of neurological diseases are aging-promoted processes. An important issue is how normal brain aging transitions to pathological aging, giving rise to neurodegenerative disorders (Wyss-Coray, 2016; Hou et al., 2019; Juan and Adlard, 2019). Despite this central role in disease pathogenesis and morbidity, the aging of the brain has not been well understood at a molecular level. Several hypotheses, such as DNA damage, loss of neural circuits and synapses, and mitochondrial dysfunction theories, were established (Lu et al., 2004; Yankner et al., 2008; Stern, 2012; Hou et al., 2019). Exploring molecular changes in the aging brain can provide a basis for a better understanding of neurodegenerative diseases and dementia.

SOM is a clustering and classification method based on neural network (Furukawa, 2009). Similar to other types of center point clustering algorithms such as K-means, SOM also finds a set of centroids (also called codebook vector), and then maps each object in the data set to the corresponding centroids according to the principle of most similarity. In neural network terms, each neuron corresponds to a center point. In our study, we performed SOM on gene expression matrix to cluster genes with highly similar expression patterns and find the pattern in which gene expression decreases with aging.

Weighted gene co-expression network analysis (WGCNA) is a biology algorithm used to describe the correlation of gene expression based on the microarray data (Langfelder and Horvath, 2008). WGCNA can be used for clustering genes with highly correlated expression, for relating the modules to phenotypes to get the most phenotypic trait-related module, and for summarizing these co-expressed gene clusters by identification of the module eigengene or hub genes. Random forest (RF) is a more advanced machine learning algorithm based on decision tree. Like other decision trees, random forests can be used for both regression and classification. In this study, we conducted RF classifier to classify the different age groups based on the gene expression matrix, then we selected the most significant genes for further analysis. Further Topological network analysis can identify the key players within modules, and thus facilitate the discovery of candidate biomarkers or therapeutic targets.

In this study, we performed machine learning and WGCNA analysis on publicly accessible transcriptome data obtained from human frontal cortex of individuals at different ages. We identified 17 co-expression modules. Through calculating the correlation coefficient between the module and age phenotype, we obtained a module of interest. Next, we disclosed the overlapping genes between differentially expressed genes (DEGs of aged group compared to young group) and genes in the module of interest. Using these overlapping genes, we conducted GO and Kyoto Encyclopedia of Genes and Genomes (KEGG) pathway enrichment analysis and further identify the central players within the module through network analysis. We concluded that *ELAVL2*, *RAP2A, RAP2B*, *KLHDC3*, and *CALM1* genes are significantly associated with aging, and may be novel biomarkers involved in neurodegeneration and dementia.

## Results

### Self-Organizing Feature Map Construction and Cluster Identification

**FIGURE 1:**
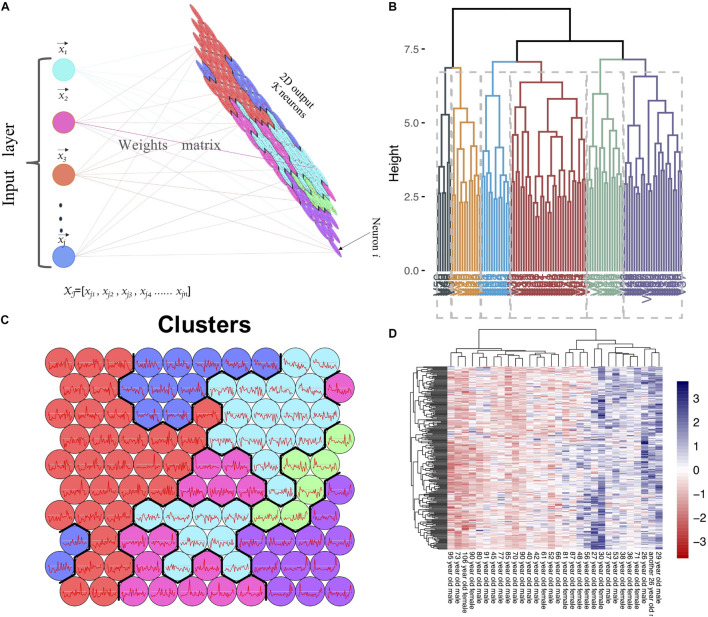
SOM clustering of genes based on microarray data. **(A)** Flow chart of SOM clustering, x_*jn*_ refers to the gene j expression level in nth sample, neuron i refers to the i cluster. **(B)** Hierarchical clustering on SOM clustering results; each 100 sub-clusters were divided into six major clusters. **(C)** The expression trend of genes in each neuron in the samples (Neuron 1–100, from bottom to top, from left to right). **(D)** The heatmap of gene expression in neuron 89, 99, and 100.

The expression matrix of GSE1572 was used as input dataset. In this dataset, after removing one abnormal sample, 30 samples were detected and used as SOM input features (Figure 1A). The expression data of each gene (in total more than 11,000 genes) in all samples was used as input data. We set the number of output neurons of the network to 100, and obtained the neural network after training (Figure 1C and Supplementary Figure 1). The weight matrix (30 × 100 size) corresponding to each feature was used as the input data of hierarchical clustering to cluster 100 neurons again. 100 neurons were clustered into six categories (Figures 1B,C). SOM clustering data showed that the gene expression of neuron 100, 99, and 89 gradually decreased with age. Next, we checked the expression levels of genes in these three clusters (Figures 1B,D). It was revealed that 240 genes, including *MAPT*, *MAP2*, *MAPK3*, *SYN2*, *RAP2A, RAP2B*, *KLHDC3*, and *CALM1*, gradually downregulated with aging.

### Weight Gene Co-expression Network Construction and Module Identification

**FIGURE 2:**
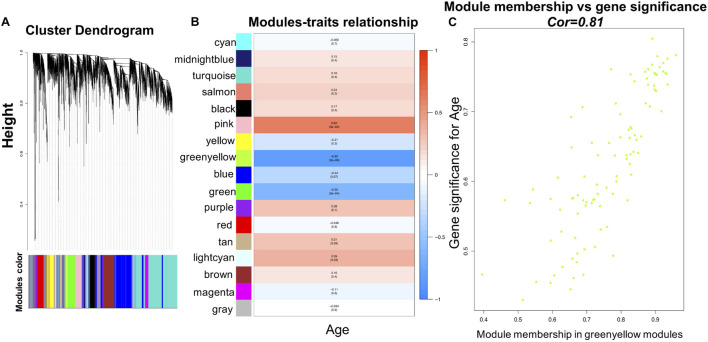
WGCNA analysis of the microarray data. **(A)** Network analysis of gene expression in aging identifies distinct modules of co-expression data. **(B)** Pearson correlation coefficient between the age and module eigengene, numbers in brackets indicate the corresponding *p*-values. **(C)** Correlation between gene significance (GS) and module membership (MM) for the clinical trait of age of genes in yellowgreen module. Cor represents absolute correlation coefficient between MM and GS.

Before WGCNA, the genes detected in GSE1572 were filtered according to the filtering procedure described in “Materials and Methods” section, and 5,000 genes were obtained. Then the 30 samples’ microarray data were read by R for Hierarchical clustering (Supplementary Figure 2A). Finally, 30 sets of data were obtained and matched to age (Supplementary Figure 2B). WGCNA was performed to identify gene co-expression networks associated with age. In the co-expression network, the degree of association between a module and other modules can be evaluated by the average connection degree and scale independence. Specifically, the closer the mean connectivity is to 0 and the closer the scale independence is to 1, the lower the correlation between modules. In the study, we set the threshold of scale independence to 0.9. We found that when the power value reaches 12, the scale independence can reach 0.9, and the mean connectivity is close to 0 (Supplementary Figure 3). Through the calculation of the correlation coefficient between genes, the genes were clustered according to the expression pattern theoretically, and the patterned genes are clustered into the same module. Seventeen co-expressed modules, ranging in size from 37 to 1,524 genes (assigning each module a color for reference), were identified (Supplementary Table 1 and Figure 2).

### Finding the Module of Interest, Functional Annotation, and Identification of the Overlapping Genes Between Differentially Expressed Genes in Young/Old Individuals and Genes in the Module of Interest Verified in Weighted Gene Co-expression Network Analysis

**FIGURE 3:**
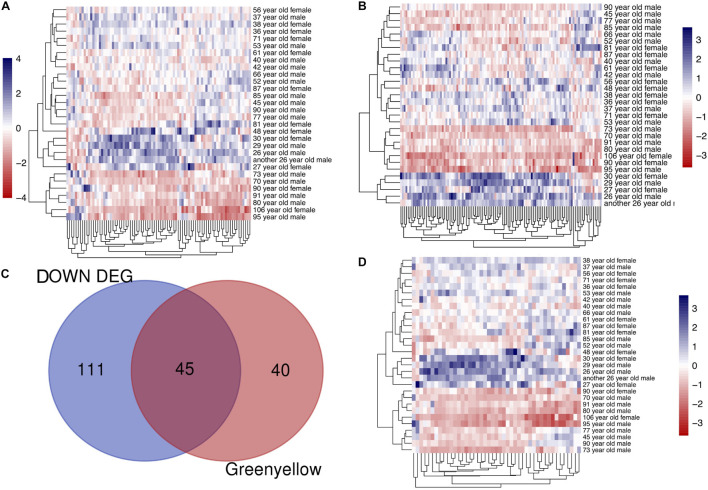
Identifying the overlapping genes between downregulated DEGs in aged group and genes in greenyellow module. **(A)** Heatmap of the expression of DEGs. **(B)** Heatmap of the gene expression in greenyellow module. **(C)** Using veen tools to find the overlap genes between downregulated genes in DEGs and genes in greenyellow module. **(D)** Heatmap showing the expression of the overlapping genes in the different samples.

To identify modules most significantly associated with age, the Pearson’s correlation coefficient between the module and age was calculated. The highest negative association in the module trait relationship was found between yellowgreen module and age score (cor = −0.83, *p* < 0.001, Figure 2B). Thus, yellowgreen module was selected as the module of interest in subsequent analyses. To confirm the correlation between module of interest and age, labeleHeatmap function was used to calculate the correlation values of module membership with gene significance (age) in the greenyellow module. The results showed significant correlation of module membership with gene significance in age (cor = 0.81, *p* < 0.0001) in greenyellow module (Figure 2C). To find the DEGs between young and aged individuals, the frontal cortical samples were grouped into individuals ≤42 and ≥73 years old and Limma packages were performed (see section “Materials and Methods” for age grouping criteria). About 4% of the genes analyzed were significantly changed (1.5-fold change or more, Figure 3A). Next, we performed overlap analysis between downregulated DEGs and genes in greenyellow module using the online veen tool; we found 45 genes in greenyellow module were also down-regulated DEGs (Figures 3B–D). These genes highly related to aging, and showed decreased expression during aging, suggesting that they might play important roles in age-related degeneration.

### Identifying Hub Genes and Gene Functional Annotation

**FIGURE 4:**
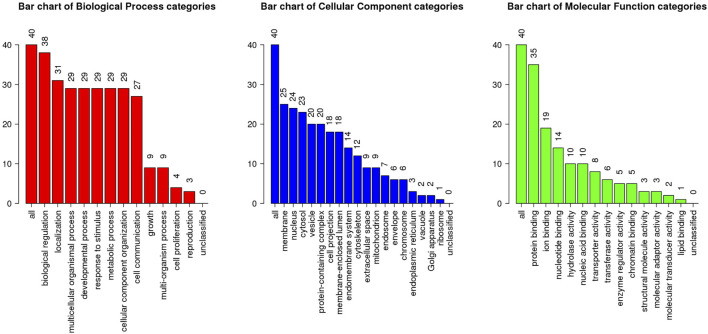
GO enrichment analysis of the overlapping genes. X-axis shows the terms of GO pathway and Y-axis shows the number of genes.

The above identified overlapping genes were subjected to GO functional and KEGG pathway enrichment analyses. Biological processes of overlapping genes were found to focus on modulation of chemical synaptic transmission and regulation of trans-synaptic signaling. Cell components of overlapping genes were found to focus on postsynaptic density and axon part; molecule functions of overlapping genes were found to focus on primary active transmembrane transporter activity and P-P-bond-hydrolysis-driven transmembrane transporter activity (Figure 4). In KEGG pathway analysis, calcium signaling pathway (*p* = 1.1498E-06; Table 1) and MAPK signaling pathway (*p* = 0.000027; Table 1) were the most significant pathways involved in overlapping genes.

**TABLE 1:** KEGG pathway analysis of the overlapping genes.

geneSet	Description	C	O	*P*-Value
hsa04020	Calcium signaling pathway	183	7	1.15E-06
hsa04014	Ras signaling pathway	232	7	5.62E-06
hsa04010	MAPK signaling pathway	295	7	2.71E-05
hsa04024	cAMP signaling pathway	199	6	2.99E-05
hsa04728	Dopaminergic synapse	131	5	5.00E-05
hsa04720	Long-term potentiation	67	4	5.49E-05
hsa05031	Amphetamine addiction	68	4	5.82E-05
hsa05161	Hepatitis B	144	5	7.86E-05
hsa04723	Retrograde endocannabinoid signaling	148	5	8.95E-05
hsa04012	ErbB signaling pathway	85	4	1.40E-04

### Identification of the Most Significant Genes and Network Construction

**FIGURE 5:**
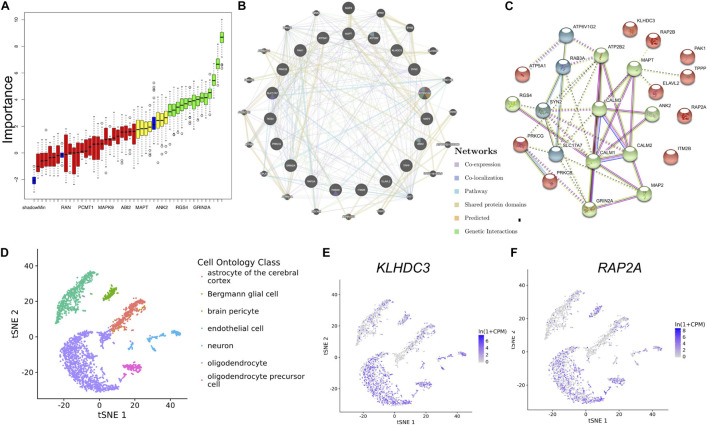
Identifying the most important genes via RF and the cellular distribution of the important genes in the brain. **(A)** Random Forest algorithm result. The blue box plot corresponds to the minimum, average, and maximum Z scores of a color attribute. The red, yellow, and green boxes represent the Z scores of rejected, tentative, and confirmed genes, respectively. **(B)** The PPI network of important genes via genemalia. **(C)** The PPI network of important genes via String. **(D)** The scatterplot shows the distribution of different kinds of cells in TSNE. **(E,F)** KLHDC3 and RAP2A expression in different cell types.

To identify the most important genes related to aging, the overlapping genes were further filtered by RF classification. Gene counts were input into RF classifier model, the unimportant genes, such as *ABI2*, *YWHAZ*, *MAPK9*, *RAN* and others were removed, and the 21 retained genes were used for the subsequent analysis (Figure 5A). To ascertain the significance of genes and analyze the network in the corresponding modules, the PPI maps were constructed via genemania and String (Figures 5B,C). Hub genes in the network, including *MAPT, PAK1, RAP2A, RAP2B, KLHDC3, TPPP*, and *ELAVL2*, were constructed. In the single-cell sequencing database Tubula, we found that the distribution of *KLHDC3* and *RAP2A* in brain cells is very similar, mainly in oligodendrocytes and neurons.

## Discussion

In this study, the dataset GSE1572 includes samples from individuals of varying age from 26 years old to 106 years old; such data from multiple samples based on age is a good candidate for SOM clustering and WGCNA analysis. First, we performed the SOM on the whole genome expression data. The SOM algorithm is usually used for data feature extraction, clustering, and classification (Furukawa, 2009). In this study, we used SOM to cluster genes in the expression matrix. In the clustering results of SOM, neurons 100, 89, and 99 are found to be related with aging. The genes in these neurons, such as *MAPT*, *MAP2*, *MAPK3*, *SYN2*, *RAP2A, RAP2B*, *KLHDC3*, and *CALM1*, were gradually down-regulated with age. Although SOM can identify some clusters of genes related to aging, this method has certain shortcomings, such as the large number of genes found, which makes it hard to screen key genes, and genes clusters having poor biological interpretation. In order to more accurately find the most relevant genes with aging, weight gene co-expression network was constructed, and we identified 17 co-expressed modules. The expression changes of genes in the same module in different samples are highly similar, indicating consistent effects and potential interaction of these gene-coded proteins in the same pathways during the aging process. Through Pearson’s correlation coefficient between the module and age, we obtained the interest module. In order to identify the significant genes, we took the intersection of the genes in the greenyellow module and the differentially expressed genes which were downregulated in aged group, and obtained 45 genes. Furthermore, we found that these overlapping genes of greenyellow module and DEGs also exist in the gene cluster found in SOM, which further confirms that these genes may be related to aging. Further KEGG pathway and GO functional enrichment analyses indicated calcium signaling pathway, long-term potentiation, and MAPK signaling pathway as the most significant pathways in the module. In order to identify genes that are most intensively related with aging, we further used one of the machine learning algorithms, Random Forest, and input the expression of the above 45 genes as feature values into the model for training, and finally screened out 21 key genes.

In another study by us (Liang et al., 2018; Chai et al., 2021), we took samples of different brain regions from different Braak stages (GSE131617) and found that microglia-mediated immune system activation plays a crucial role in the early stages of Alzheimer’s disease. The samples we used in this study are only samples of the frontal cortex of different ages, and do not contain any clinical diagnosis and pathological changes, which is more conducive to discovering the changes in the brain during the aging process.

Analysis of hub genes showed that *SYN2* might play an important role in aging. In the Cell Component (CC) enrichment analyses, postsynaptic density and distal axon were identified as the most significant CC in the network. In the Biological Process (BP) enrichment analysis, synaptic vesicle localization was revealed to be a significant BP in the network. *SYN2* is a multigene family coding synaptic vesicle (SV) phosphoproteins implicated in the regulation of synaptic transmission and plasticity (Luk et al., 2012). In previous studies, it was shown that *SYN2* knockdown mice display emotional and spatial memory deficits that aggravated during aging (Corradi et al., 2008; Boido et al., 2010). In the co-expression network constructed in the present study, the expression of *SYN2* decreases with the increase of age. We suspected that the decreased expression of *SYN2* is either a result of synapse impairment/loss during aging, or an upstream factor that induces synaptic dysfunction.

In the co-expression network, *MAPT* and *MAP2* were identified as hub genes. *MAPT* encodes microtubule-associated protein tau, which promotes the stability and assembly of microtubules in axon of neurons (Dehmelt and Halpain, 2005; Irwin et al., 2013; Wang and Mandelkow, 2016; Saha and Sen, 2019; Vogels et al., 2019). This was in accordance with the fact that distal axon is a significant CC in the GO enrichment analysis. In age-related tauopathy, tau pathology has been considered as a significant marker in neurodegeneration. *MAP2* gene encodes dendritic marker MAP-2, which is also a microtubule-associated protein (Friedrich and Aszódi, 1991; Dehmelt and Halpain, 2005). Microtubule is a key player in neuronal activities and axoplasmic flow under physiological conditions. In our study, we found that with the increase of age, the expression of *MAPT* and MAP2 decreases, which may be a result of neurite degeneration during aging. However, genes that code other skeletal proteins such as tubulin were not identified as hub genes in aging. This result indicates that microtubule-associated proteins tau and MAP-2 may participate in aging-related pathogenesis through mechanisms other than cell skeletal stability.

Analysis of hub genes also showed that *RAP2A* and *RAP2B* were hub genes in the co-expression network. RAP2A and RAP2B belong to the small GTPase superfamily (Emery et al., 2017). Most studies about RAP2A and RAP2B focus on their functions in tumor (Zheng et al., 2017; Zhang et al., 2020). RAP2A is overexpressed in a multitude of human cancers and plays an important role in cytoskeleton rearrangement, arteriogenesis, and cell migration. In neurons, it was found that RAP2 stimulated dendritic pruning, reduced synaptic density, and caused removal of synaptic AMPA receptors, suggesting that RAP2 plays a role in regulating synaptic functions (Kawabe et al., 2010; Hu et al., 2019). In our study, we found that *RAP2A* and *RAP2B* were interacted and co-localized with *MAP2* in the co-expression network and string network. Therefore, RAP2A and RAP2B may have a similar function or cooperate with MAP2. We speculate that the main function of RAP2A in the brain is also involved in regulation of dendritic development and plasticity.

To our surprise, *KLDHC3* was found mainly co-expressed with *RAP2A* and *RAP2B* in the co-expression network. Its related pathways are Unfolded Protein Response (UPR) and metabolism of proteins, and a few studies report its function in the brain (Niculescu et al., 2015). In our study, *KLHDC3* and *RAP2A* are consistently distributed in different cells in the brain (Figures 5D–F), so we speculate they may also participate in similar functions in the brain. The decrease of the expression of *KLHDC3* with age may also play a role in the impairment of dendritic and synaptic plasticity during aging. Further studies needed to reveal the function of KLDHC3 in neurons.

At last, *ELAVL2* was characterized as a hub gene with *PAK1*, *MAPT*, *RAP2A*, and *RAP2B* in the same module. Some studies report that ELAVL2-regulated pathways are involved in normal human brain function and their disruption may play a role in neurodevelopmental disorders such as autism spectrum disorder (ASD) (Berto et al., 2016; Ohi et al., 2017; Kato et al., 2019). However, the function of ELAVL2 in the aging brain has not been reported yet. In our study, ELAVL2 was found to be co-localized with PAK1, and co-expressed and interacted with tau. Both tau and PAK1 are involved in axonal guidance and neuronal migration (Dehmelt and Halpain, 2005; Koth et al., 2014). Therefore, we speculate that ELAVL2 may play a consistent role with tau and PAK1 in neurons.

In summary, through machine learning and WGCNA on microarray data from human frontal cortex, we uncovered that *RAP2A, RAP2B, KLHDC3*, and *ELAVL2* may be associated with aging. The proteins encoded by these genes may play a coordinated role in the brain with the proteins tau, MAP-2, SYN, and CALM family in neurodegenerative diseases, which may be novel biomarkers of neurodegenerative diseases caused by aging.

## Materials and Methods

### Data Acquisition and Preprocessing

The data used in this paper was obtained from the GEO database in NCBI^1^ (Gene Expression Omnibus), and the data entry number is GSE1572 (Lu et al., 2004). The platform is Affymetrix Human Genome U95 Version 2 Array [HG_U95Av2]. Gene expression in the frontal cortex of 18 normal males and 12 normal females at 26–106 years old was detected. The normalized data was downloaded and the expression matrix was obtained, and data filtering was performed before WGCNA analysis. For data filtering, the standard deviation of the gene expression was calculated to obtain a list with decreasing standard deviations, the first 5,000 genes with large standard deviations were obtained, and the probe without corresponding annotation information were removed. There were about 11,000 genes in the dataset; after the data preprocessing, we kept 5,000 genes for further analysis.

### Finding Genes With Highly Similar Expression Pattern Through Self-Organizing Feature Map Algorithm

The SOM clustering was constructed by kohonen package based on R 3.4.2 (Furukawa, 2009). The 31 frontal cortical samples were treated as 31 input features. The expression counts of each gene in 31 samples are used as input data. Through inputting the data to SOM cluster model to cluster the genes, we can obtain the cluster to show which gene expression decreases with aging.

### Construction of Weighted Gene Co-expression Network and Identification of Significant Modules

Data was processed using R 3.4.2 software. To ensure that the results of network construction are reliable, abnormal samples were removed. Then, the weighted gene co-expression network was constructed by WGCNA package based on R 3.4.2. First, the Pearson correlation coefficient was calculated to assess the similarity of the gene expression profiles. Second, the correlation coefficients between genes were weighted by a power function to obtain a scale-free network. A gene module is a cluster of densely interconnected genes in terms of co-expression. Then, hierarchical cluster was used to identify gene modules and different modules were represented by different colors. Dynamic treecut method was used to identify different modules, the adjacency matrix was converted to a topology overlay matrix (TOM), and modules were detected by cluster analysis during module selection.

### Correlation Analysis of Gene Modules With Clinical Phenotype

To detect the associations of modules to clinical phenotype (age), first, the age data and gene expression data were correlated using the match function. Secondly, the associations of the module eigengene (ME) to the age were calculated by Pearson’s correlation analysis. Modules showing significant association to age were obtained. At last, to further confirm the modules with significant correlation to age, the correlation coefficient between the module membership (gene expression level) with gene significance (GS, for assessing the association of genes with phenotypes) was calculated using the labeleHeatmap function, and the *p*-values were obtained.

### Finding the Overlapping Genes Between the Differentially Expressed Genes (DEGs in Aged Compared to Young Group) and Genes in the Module of Interest Verified by Weighted Gene Co-expression Network Analysis

The frontal cortical samples were grouped into individuals ≤42 (young group) and ≥73 years (aged group) and Limma packages were performed to find the DEGs; the group of individuals ≤42 years old showed the most homogeneous pattern of gene expression, and the group ≥73 years old was also relatively homogeneous. Moreover, these two age groups were negatively correlated with each other. In contrast, the middle age group ranging in age from 45 to 71 exhibited much greater heterogeneity, with some cases resembling the young group and others resembling the aged group (Lu et al., 2004; Ritchie et al., 2015). Next, the overlapping genes between downregulated DEGs and genes in the module of interest were discovered by using online veen tools.^2^

### Gene Ontology and Kyoto Encyclopedia of Genes and Genomes Pathway Enrichment Analyses, Identification of Hub Genes, and Protein-Protein Interaction Analysis

For the obtained overlapping genes, functional enrichment of Gene Ontology (GO) and KEGG pathways analyses were performed using GSAT (Zhang et al., 2005)^3^ and GOplot packages based on R3.4.2. *P*-value < 0.05 was considered to be significant enrichment. These genes were also analyzed using cytoHubba in Cytoscape for identification of hub genes. The identified hub genes were further confirmed and analyzed using genemania (Warde-Farley et al., 2010).^4^ String network was constructed by the online tools String.^5^

### Application of Random Forest Algorithm to Find the Most Important Genes Related to Aging

The frontal cortical samples were grouped into individuals ≤42 (young) and ≥73 years (old). Through inputting the overlapping genes counts into random forest classifier model to predict which group the samples belong to, the most important overlapping genes for the most accurate model for grouping were identified.

### Exploring the Cellular Distribution of the Identified Genes

By using the single cell RNA-seq database Tubula^6^ (Tabula Muris Consortium et al., 2018), the cellular distribution of the identified important genes were further explored.

## Data Availability Statement

The datasets presented in this study can be found in online repositories. The names of the repository/repositories and accession number(s) can be found below: https://www.ncbi.nlm.nih.gov/geo/query/acc.cgi?acc=GSE1572.

## Author Contributions

KC contributed to the study design, performed the experiments, and contributed to the writing of the manuscript. JL contributed to the study design and the writing of the manuscript. XZ, PC, SC, WY, HG, RL, and WH conducted the experiments. CP, GL, and DS provided critical devices and contributed to the study design. All authors read and approved the final manuscript.

## Conflict of Interest

The authors declare that the research was conducted in the absence of any commercial or financial relationships that could be construed as a potential conflict of interest.

## Publisher’s Note

All claims expressed in this article are solely those of the authors and do not necessarily represent those of their affiliated organizations, or those of the publisher, the editors and the reviewers. Any product that may be evaluated in this article, or claim that may be made by its manufacturer, is not guaranteed or endorsed by the publisher.

## Footnotes

1
https://www.ncbi.nlm.nih.gov/geo/query/acc.cgi?acc=GSE1572

2
http://bioinformatics.psb.ugent.be/webtools/Venn/

3
http://www.webgestalt.org/option.php

4
http://genemania.org

5
http://string-db.org

6
https://tabula-muris.ds.czbiohub.org
