# Supplementary material for: Application of Machine Learning and Weighted Gene Co-expression Network Algorithm to Explore the Hub Genes in the Aging Brain
Source: Front Aging Neurosci. 2021 Oct 18;13:707165. doi: 10.3389/fnagi.2021.707165 (PMC8558222; doi:10.3389/fnagi.2021.707165)
Supplement: Supplementary file 1 [file Data_Sheet_1.DOCX]

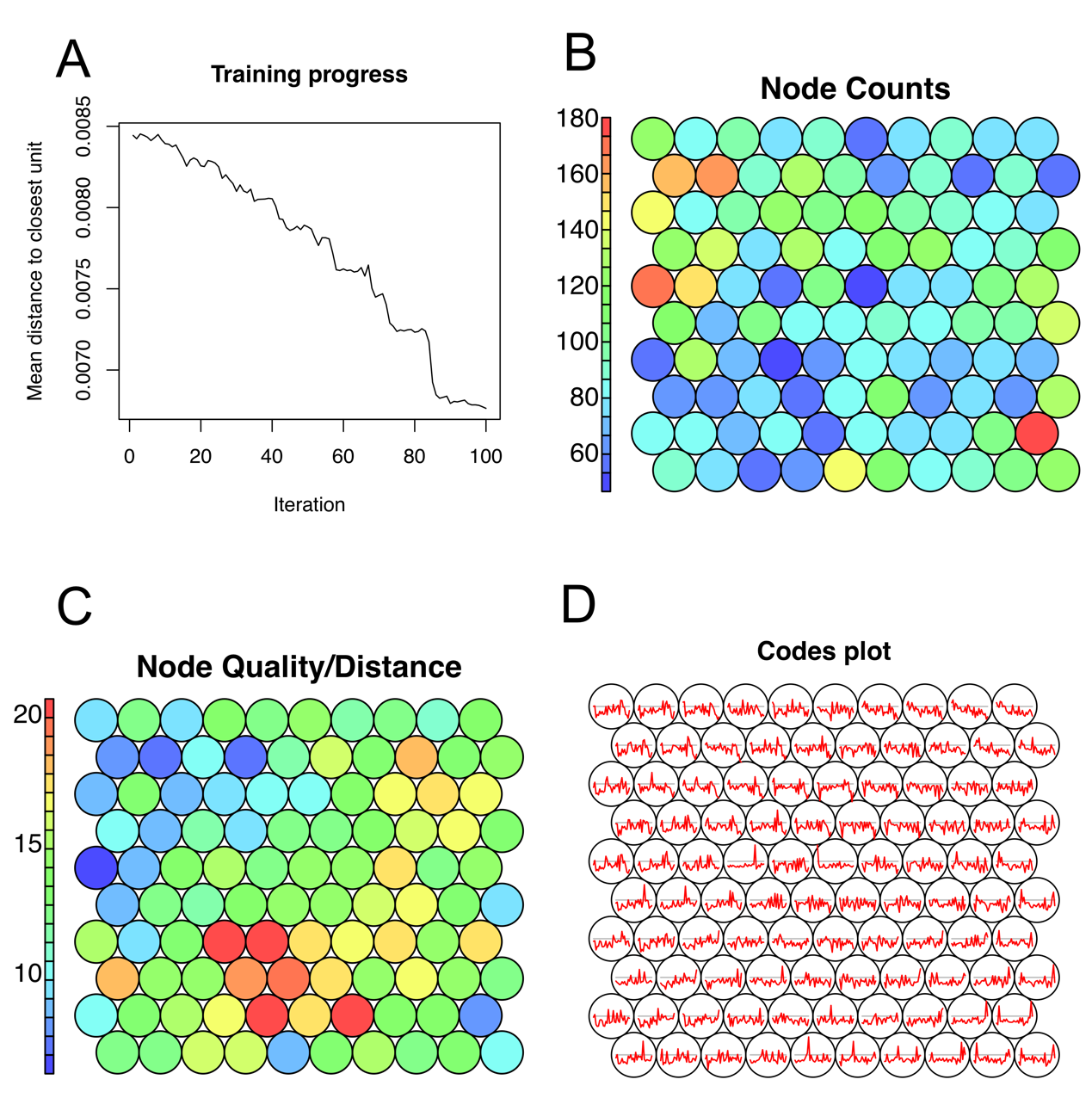


**Figure.S1. The training and cluster of SOM.**  **A)** Plot of the training progress – how the node distances have stablished over time. **B)** The number of genes mapped to the individual units.

**C)** Map quality. The codebook shows the mean distance of objects mapped to a unit to the codebook vector of that unit. **D)** The codebook shows the genes trend in each unit .


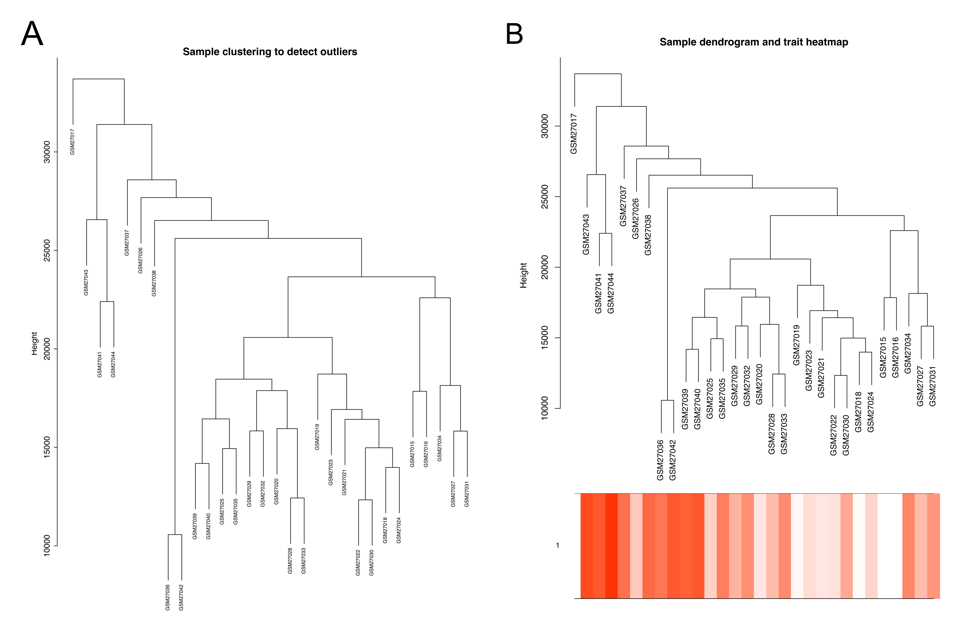


**Figure.S2. Dendrogram of the samples cluster**. **A)** Hierarchical clustering of the samples based on the expression matrix. **B)** Hierarchical clustering of samples with age, orange heatmap represent for the different age.


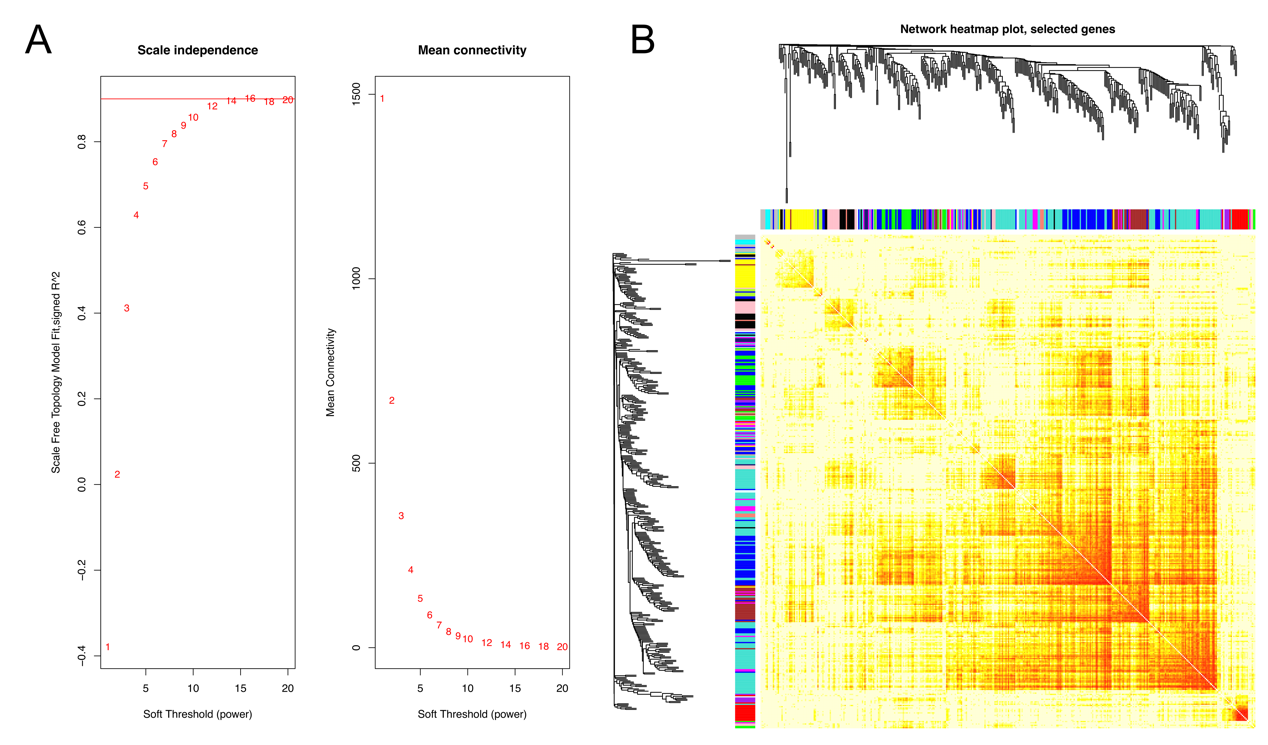


**Figure.S3. Selection of soft threshold and network construction** . **A)** The scale independence and mean connectivity analysis showed that when the weighted value equals to 12, the average degree of connectivity was close to 0, and scale independence was greater than 0.9. **B)** The heatmap of Module correlation matrix, each color represent one module.


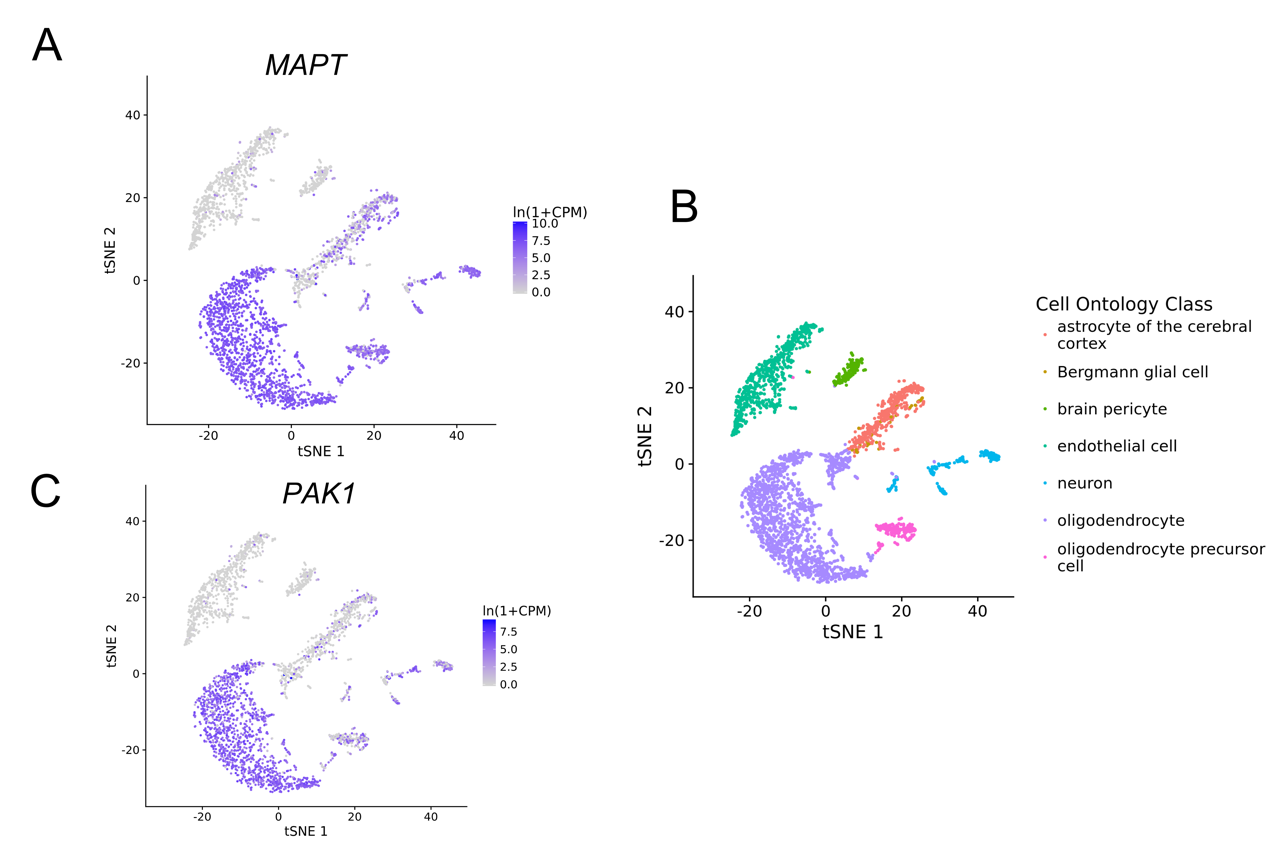


**Figure.S4. The scRNA-seq data dimensionality reduction via TSNE. B)** The scatterplot show that the different kind cell distribution in TSNE. **A,C)** MAPT and PAK1 expression in different kind of cell.

| turquoise | blue | brown | yellow | green | red | black | pink | magenta |
| --- | --- | --- | --- | --- | --- | --- | --- | --- |
| 1524 | 907 | 389 | 266 | 262 | 223 | 214 | 209 | 194 |
| purple | grey | greenyellow | tan | salmon | cyan | midnightblue | lightcyan | |
| 132 | 106 | 97 | 63 | 56 | 46 | 44 | 37 | |

**Table S1**
Module and the number of genes in each module
